# 

**Published:** 1893-11-11

**Authors:** 


PRICE TWOPENCE.

No. 372, Vol. XV.—Nov. 11th, 1893.

[Registered at the General Post Office as a Newspaper for transmission in the United Kingdom and Abroad.]

WITH EXTRA NURSING SUPPLEME

Per Annum, 8s. 6d., post free.

Monthly Parts, 6d.; post free, 8d.

Ditto per Annum, 8s., post free.

### THE HOSPITAL

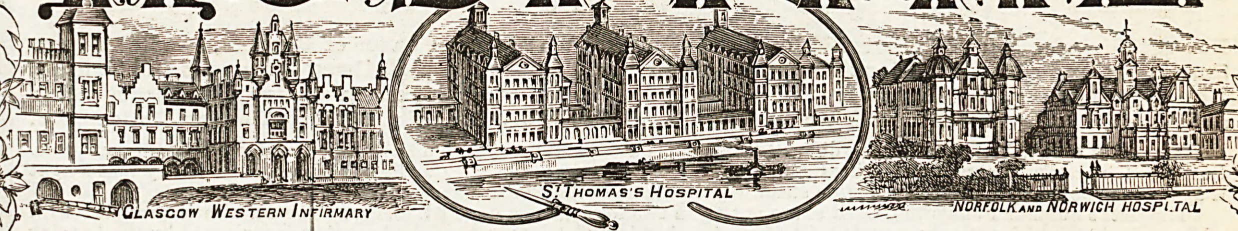

WITH EXTRA NURSING SUPPLEMENT.
No. 372. Vol. XV. SATURDAY, NOVEMBER 11TH, 1893. [TWOPENCE.

